# Socioeconomic status and sex ratio in the contemporary Hungarian population

**DOI:** 10.1017/ehs.2024.39

**Published:** 2024-10-30

**Authors:** Fanni Sarkadi, Eszter Szász, Balázs Rosivall

**Affiliations:** 1 Behavioural Ecology Group, Department of Systematic Zoology and Ecology, Institute of Biology, ELTE Eötvös Loránd University, Budapest, Hungary; 2 Doctoral School of Biology, Institute of Biology, ELTE Eötvös Loránd University, Budapest, Hungary

**Keywords:** offspring sex ratio, sex ratio adjustment, Trivers–Willard hypothesis, reproductive success, socioeconomic status

## Abstract

According to the Trivers–Willard hypothesis (TWH), when the mother's condition around conception influences the future reproductive success of male and female offspring differently, the adjustment of offspring sex ratio (SR) to maternal condition will increase the parents’ fitness. The TWH has been tested in several taxa, including humans where socioeconomic status as an index of condition has been widely used. The results are inconsistent, possibly because the preconditions of the TWH are not always met. To investigate the preconditions and prediction of the TWH in the contemporary Hungarian population, we collected data by an online questionnaire on self-perceived childhood living standard, the number of children and the sex of the respondents’ siblings. We found no sex-specific relationship between reproductive success and childhood living standards, thus the precondition of the TWH was not met. We found no relationship between socioeconomic status and offspring SR when data from the whole country was used, but there was a tendency in the predicted direction when we used data from Budapest and considered the SR of only those family members who were born under similar conditions. Similar approaches should be preferred in the future to avoid noise caused by changing status during the reproductive lifespan.

## Graphical abstract

**Figure:**
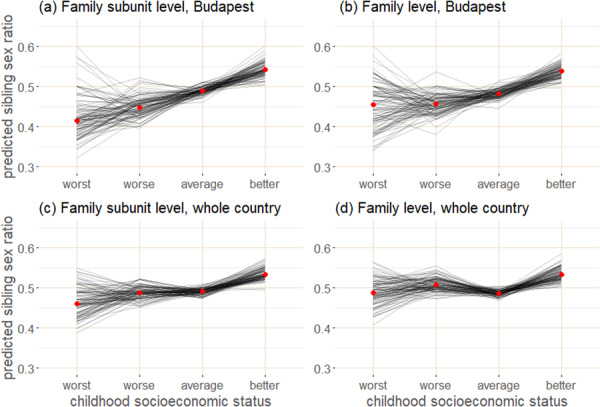

**Social media summary:** Relative socioeconomic status does not predict children sex ratio in contemporary Hungary.

## Introduction

Since its publication 50 years ago, the hypothesis of Robert L. Trivers and Dan E. Willard has become the most cited and widely tested in the field of sex allocation research (Navara, 2018a). The Trivers–Willard hypothesis (hereafter TWH; Trivers & Willard 1973) assumes that (i) the mother's condition during parental investment positively correlates with the condition of her offspring at the end of parental investment, (ii) the differences in condition of offspring are maintained into adulthood, and (iii) condition in adulthood has a stronger effect on the reproductive success of males than that of females, since the reproductive success of males may vary more with status categories and can potentially be higher than that of females. If the assumptions are met, then mothers in better condition are expected to produce male offspring in a higher proportion, while mothers in worse condition will benefit from producing proportionally more female offspring. Although Trivers and Willard (1973) developed their hypothesis with socially polygynous mammals in mind, its assumptions may be valid in other species too.

The central trait in the TWH was originally body condition (that can be measured e.g. by weight; Trivers & Willard 1973). However, the TWH can be extended to any trait that can be passed down by the parent to the offspring and has sex-specific effects on the future reproductive success of the offspring. Consequently, the prediction of the hypothesis has been extensively tested not only with absolute body condition (Cameron & Linklater, 2000; Whittingham & Dunn, 2000), but also for example, with relative condition (Douhard et al., 2016), social rank (Clancey & Byers, 2016; Clutton-Brock et al., 1984) and mass (Servanty et al., 2007; Toni et al., 2021), or tail loss of lizards (S. F. Fox & McCoy, 2000). Surprisingly, an extension of the TWH has been applied to plants as well (Freeman et al., 1994). However, the results of this large body of research are mixed across and within species (Cameron, 2004; Douhard, 2017; Hewison & Gaillard, 1999; Sheldon & West, 2004; Thouzeau et al., 2023).

The TWH has been frequently tested also on humans with a shift from using physical characteristics as a proxy for maternal condition to using socioeconomic status (hereafter SES) as central trait, as suggested by Trivers and Willard (1973). However, Trivers and Willard (1973) did not define SES. Hence different SES measures and indicators have been used in the studies of the TWH. For example, income (Kolk & Schnettler, 2016), wealth (Cameron & Dalerum, 2009; Schnettler, 2013), place or ownership of residence (Houdek et al., 2020; Wallner et al., 2012), educational attainment (Almond & Edlund, 2007) and occupation (Kolk & Schnettler, 2016; Schacht et al., 2019) have been used in former studies as proxies for SES.

Human studies on offspring sex ratios have yielded mixed results (Houdek et al., 2020; James, 2012; Kolk & Schnettler, 2016; Lazarus, 2002; Navara, 2018b). There are several possible sources of the inconclusive support for the TWH in humans. First, it is unknown which indicator of SES (see above) matters most as a proxy for condition (Kolk & Schnettler, 2016). Furthermore, the sex ratio of children is likely to be influenced by the mother's relative rather than absolute condition (i.e. relative to the condition of other mothers in the population during reproduction) (Douhard, 2017; Lazarus, 2002; Trivers & Willard, 1973). This is especially true when one studies multiple cohorts or multiple (sub)populations simultaneously; however, studies vary in whether they use relative or absolute indicators.

Finally, although the prediction of the TWH is widely tested, the assumptions of the TWH are often overlooked and have only been investigated in a minority of studies (Douhard, 2017; Schindler et al., 2015). Testing the assumptions on the studied population is crucial because when the assumptions are not valid in the population under study, then the validity of the TWH cannot be tested. In many cases, validity of assumptions was inferred based on results of previous studies, because the source of the data (e.g. censuses or population registers) did not allow the assumptions to be directly tested. In contemporary societies the assumption regarding heredity of status is often supported; however, the results on the sex-specific positive relationship between fertility and socioeconomic status are equivocal (see Kolk & Schnettler 2016 for references), which makes testing this assumption more important.

In this paper, we tested whether the assumptions *and* the prediction of the TWH hold for the Hungarian contemporary population by conducting a retrospective questionnaire which was specifically designed for gaining data on the sex ratio and reproductive success of all siblings within a family. As an estimator of SES, we used relative living standard of the respondents during their childhood. First we tested the model's preconditions together in a single model. Namely, using data of respondents who are unlikely to have more children, we investigated whether the respondent's relative living standard during childhood is associated with reproductive success in the two sexes differently. Then, we tested whether a positive relationship between relative living standard in childhood and the sex ratio of full siblings within a family exists (hereafter *prediction*). We must note that studies on animal sex ratio adjustment most often investigate brood sex ratio patterns; that is whether the sex ratio of offspring born at the same time (i.e. under the same conditions) is related to certain traits (e.g. body condition of the mother). However, human studies generally investigate family sex ratios that is the sex ratio of successive offspring produced over a long period of time, often the whole lifetime (e.g. Li et al., 2022; Sorokowski et al., 2019) during which the condition of the mother may have changed considerably**.** If we expect that the sex of the offspring is influenced by the actual condition of the mother, studies using a single measure of condition and whole family sex ratios may fail to find the relationship even if it exists. Therefore, we investigated not only whole family sex ratios, but also the sex ratios of family subunits (i.e. offspring presumably born under similar conditions; see Methods).

## Methods

### Data collection

We collected data via an online questionnaire from June 2019 to August 2020. Participation was completely anonymous, data were secured and the project gained prior ethical permit from the Institute of Biology, Eötvös Loránd University (reference number TTK/6006/2(2019)).

We targeted people who had presumably completed their reproduction (over age 45 for women and age 55 for men, see below) and had siblings. Originally, we aimed to recruit participants from Budapest, and tried to contact all state-owned secondary schools’ headmasters to spread the questionnaire among the parents. Owing to the low response rate, later we submitted a press release through Eötvös Loránd University, and we conducted a paid Facebook campaign by specifically targeting people over age 40, thus finally, we received responses from all parts of Hungary.

The questionnaire consisted of three pages, of which the third page contained sensitive questions (e.g. questions on use of induced abortion), hence it was set to be facultative. For the translation of the questions, see Supplementary Table 1. Overall, 3791 adult individuals filled out at least the first two pages, and only 148 of them did not answer the question on induced abortion. The overall participation of men (*N =* 715) and women (*N =* 3076) was uneven: women were more willing to fill out the questionnaire. This is a common pattern in online survey responsiveness (Slauson-Blevins & Johnson, 2016; Smith, 2008).

After filling out the questionnaire, every respondent received a unique code, and they were asked to share the link to the questionnaire and their code with their siblings. In this way, we could identify respondents who belonged to the same family and gave them the same family code (*N =* 530 respondents). However, we received a few responses with erroneous family code (*N =* 16). In addition, it is also possible that someone forgot to share the family code with his/her siblings or siblings filled in the questionnaire independently without knowing the family code of each other. To overcome these issues, we looked for possible family ties in the dataset by searching for correspondence between the respondents’ number of siblings, the year and place of birth, and number of children (excluding foetuses with unknown sex). We tested this method on a subset of data that included only respondents linked to families by the unique codes. With this method we found circa 90% of the real families, and all of the found families were correct. This means that it is unlikely that false families were found in the whole dataset using our algorithm, but the whole dataset might still contain some respondents who belonged to families but were handled in the dataset as independent respondents. With the applied method we found 47 additional respondents that could be linked to a family.

### Variables

To quantify the childhood socioeconomic status of the participants, we asked the participants to recall and score their family's standard of living from their birth to 10 years of age. If their standards had changed considerably during this period, we asked them to recall the earliest period they remember. Childhood living standard was defined on a scale from 1 to 5, where 1 = had a very hard time, 2 = lived worse than average, 3 = lived at an average standard, 4 = lived better than average and 5 = lived very well.

To quantify the sex ratio in the participants’ family, we asked them about the number and sex of their full siblings. However, the birth interval between siblings can be so large that the siblings may not share the same childhood living standards. To control for this, we defined ‘family subunit sex ratio’ as well: the sex ratio of the part of the family, where the age gap between successive siblings is maximum 5 years, the family's financial situation has not changed substantially between the births of the siblings according to the respondent, and it contains at least one respondent. If multiple family subunits existed within a family, the largest subunit was used, or if they were of the same size, a random selection was made. Single-member subunits occurred when the largest/single subunit contained only one member of the family (i.e. the respondent). In case of families where all of the successive siblings were born within 5 years and the financial situation of the family did not change between their births, family subunit sex ratio and family sex ratio were the same. Note that we present the sex ratio in this paper as the number of male siblings divided by the number of all siblings within a family/family subunit.

Respondents were asked to give the total number of biological children they had, and separately the number of sons, daughters and foetuses of unknown sex as well, to make sure they had typed the numbers correctly. Reproductive success was defined as the total number of the respondent's children including foetuses with whom the respondents (or their partner) were pregnant at the time of the survey.

To control for the potential noise caused by variation in the living environment we asked respondents about their settlement. They could choose from the following five categories: village (<5000 residents), small town (5000–20,000), medium-sized town (20,000–100,000), large town (>100,000) and Budapest (the only metropolis of Hungary). Respondents were asked to provide the county and settlement type of their residence during childhood and adulthood. Collecting more detailed information on residence was not possible, otherwise this could have compromised the anonymity of respondents given the other information they provided about themselves. Data on the highest education level of the respondents and their parents were also collected, and used to validate the use of our estimate of childhood SES (for details of the sample size, analysis and results, see Supplementary Information 1), because educational attainment has been shown to fundamentally influence material well-being (reviewed by Edgerton et al., 2012).

### Testing the precondition – data selection and analysis

To eliminate the differences in the socioeconomic environment across countries, we restricted our data to respondents who were born in Hungary, which was the vast majority of the respondents (3649 out of the 3791). Data of respondents with missing information on childhood residence (*N =* 7) were omitted. We also excluded those respondents who currently lived abroad, or where we had missing information on their residence (*N =* 116).

To test the precondition of the TWH, we used the data of only those who could be regarded as having had final reproductive success (*N =* 2429), i.e. the respondent was surely at least 45/55 years old (women/men, respectively, calculated from year of birth and year of filling out the questionnaire) and did not plan to have more children (i.e. did not answer ‘yes’ to the following question: Would you and your current partner like to have more children?); or the respondent was up to 10 years younger but claimed to be in a stable relationship (i.e. answered ‘yes’ to the following question: Do you think you and your partner will still be together in 10 years?) and answered explicitly not planning to have more children. For information on reproductive timing in Hungary, see Supplementary Information 2.

In line with the definition, our reproductive success estimate also contained foetuses with whom the respondents (or their partners) were pregnant; however, only eight of the 2429 respondents were expecting a child at the time of completing the questionnaire. In the rare case where the number of children was not equal to the sum of daughters, sons and foetuses reported by the respondent, the respondent was excluded from the database owing to data inconsistency (*N =* 5).

To avoid pseudoreplication, we used only one set of data per family in all analyses. From the 113 families with multiple respondents, we removed 122 respondents. We retained the respondent from each family whose reproductive success was certainly not affected by induced abortion (this was an important criterion when testing the precondition, see below) and was male (males were preferred because they were underrepresented in the dataset; see above). If these were true for more than one respondent, or if multiple respondents from a family could have been affected by induced abortion and were all from the same sex, we chose at random (*N =* 52 families). After controlling for pseudoreplication, our dataset consisted of 2307 responses from the whole country that met these requirements.

**Figure 1.**
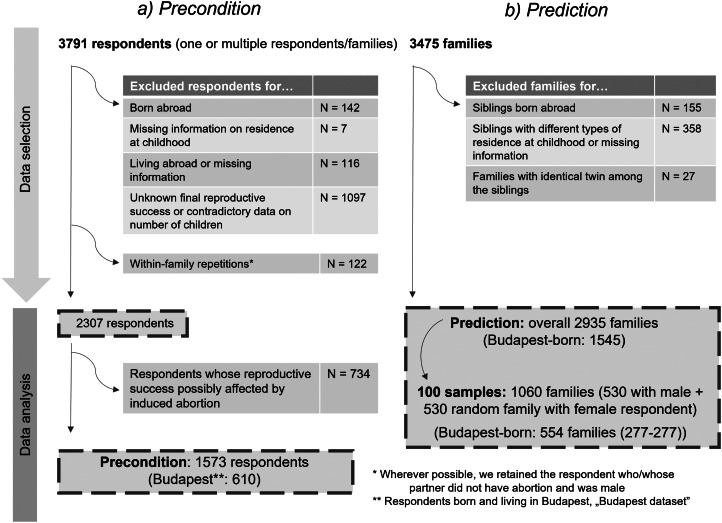
Flow chart describing data selection. The exclusion criteria appear in the order in which they were applied during data selection.

Since the use of induced abortion might decrease the number of children one has, we tested the precondition on subsets of our dataset, where final reproductive success of the respondents was certainly not affected by induced abortion (i.e. answered ‘no’ to questions Q3.25 and Q3.26 in Supplementary Table 1; ‘whole country dataset’, *N =* 1573, Figure 1a). To reduce noise caused by spatial heterogeneity we repeated the analyses on data from respondents born and living in Budapest (‘Budapest dataset’, *N =* 610, Figure 1a). For an overview of the exclusion/selection criteria, see Figure 1a.

We built a generalised linear model (GLM) with log link and Poisson error distribution adjusted to underdispersion (using function ‘glm’ with family attribute ‘quasipoisson’ from R package ‘stats’; R Core Team, 2023). The response variable was the respondent's number of children. The explanatory variables were childhood SES (ordinal variable, with Helmert contrast setting, which compares each level of the ordinal categorical variable with the mean of subsequent levels), the sex of the respondent (categorical, ‘contr.sum’ setting) and the two-way interaction of these two variables. The two highest childhood SES categories were combined, because only seven respondents reported as living very well during childhood, and it resulted in poor model diagnostics. Hence, the analysed four SES levels are abbreviated as ‘worst’, ‘worse’, ‘average’ and ‘better’ in the tables and figures. After we considered the potential confounding variables, in the GLM we also controlled for the number of siblings within the family, because this might lower the relative status one experienced during childhood, and because a positive association has been found between the family size of parents and children (Beaujouan & Solaz, 2019). Also, in case of the analysis of the ‘whole country dataset’, settlement type in childhood was added to the model as a categorical control variable (with the ‘contr.sum’ setting), because fertility might be related to settlement size (Kulu et al., 2007), hence it is expected to be associated with number of siblings and, through settlement type in adulthood, with number of children as well. Fits of the models were validated visually by plotting the model residuals against the predicted values (using function ‘plot’ from R package ‘stats’; R Core Team, 2023) and using binned residuals plots (using function ‘binnedplot’ from R package ‘arm v1.13’; Gelman & Su, 2022). To improve model fit, the single respondent with an outstanding number of children (13) was removed from the dataset, so the final sample size for the ‘whole country dataset’ was *N =* 1572. We applied type 3 Wald chi-square test statistics (using the function ‘Anova’ from R package ‘car v3.1’; J. Fox & Weisberg, 2019). We used the function ‘emmeans’ from the package ‘emmeans v1.10.0’ (Lenth, 2024) for the *post-hoc* comparisons with false discovery rate *p*-value correction.

### Testing the prediction – data selection and analysis

Originally, we had information about 3475 families (and family subunits; see above). To eliminate the differences in the socioeconomic environment across countries, we removed those families where any of the siblings within a family (including the respondent) was not born in Hungary (*N =* 155). We also removed those families where the type of residence during childhood differed among the siblings, or information on type of childhood residence for any of the siblings had not been provided (*N =* 358). Also, families where identical twins were born among the siblings (*N =* 27) were excluded, because the sex of identical twins cannot be considered independent, and therefore identical twins bias the sex ratio data. As in the remaining dataset there were more female than male respondents (families with male and female respondents in the ‘Budapest dataset’ (i.e. where every sibling in the family was born in Budapest): 277 + 1268; in the ‘whole country dataset’: 530 + 2405) and the mean number of siblings was around 2 (2.12 ± 0.86 and 1.74 ± 0.78 in the ‘Budapest dataset’; 2.16 ± 0.84 and 1.76 ± 0.79 in the ‘whole country dataset’; for families and family subunits, mean ± SD), the family and family subunit sex ratios were also strongly female-biased, which resulted in poor model diagnostics after running the GLMs. To overcome this issue, we randomly selected 277 and 530 female respondents from the ‘Budapest’ and the ‘whole country’ datasets respectively and merged these data with the male respondents’ data, hence the overall sample size was *N =* 554 and 1060 respectively (for an overview of the exclusion criteria and sample sizes, see Figure 1b). The random sampling and the following analysis (GLM, see below) were performed 100 times.

The TWH predicts a positive relationship between SES and offspring sex ratio. We built GLMs with binomial error structure and a logit link function (using the function ‘glm’ from R package ‘car v3.1’; J. Fox & Weisberg, 2019). The response variable was the number of sons, and the binomial denominator was the number of offspring, either in the family or in the family subunit. The explanatory variables were childhood SES (ordinal categorical variable, with Helmert contrast, see above), and in case of the analysis of the ‘whole country dataset’, type of settlement (as a categorical control variable, with ‘contr.sum’ setting, for similar reasons as in the case of the precondition model). Owing to the low number of respondents who belonged to the highest relative SES category the GLMs on some random samples did not run, and in the other models the estimates for the SES contrasts were unreliable with high standard errors; therefore we treated the two highest SES categories as one just like in the case of the precondition. We report the median *p*-values of the type 3 Wald chi-square tests for SES (using the function ‘Anova’, see above) and the *p*-value and estimate of the contrast of the second and fourth SES categories (‘worse than average’ – ‘better than average’) using ‘emmeans’. We used the second and not the first category (i.e. ‘had a very hard time’) as the sample size of the first category for the whole dataset was much smaller than for the second and fourth categories (see Supplementary Table 2).

We performed all statistical analyses and data visualisations in R version 4.3.2 (R Core Team, 2023). The following packages were used for data visualisation: ‘ggplot2 v3.5.1’ (Wickham, 2016), ‘patchwork v1.2.0’ (Pedersen, 2024), ‘ggeffects v1.5.0’ (Lüdecke, 2018) and ‘marginaleffects v0.18.0’ (Arel-Bundock, 2024).

## Results

### Descriptive statistics for the variables used in the analyses

Tables 1 and 2 and Supplementary Tables S2 and S3 contain sample sizes and descriptive statistics for the variables used. The median birth year of the respondents from the whole dataset for the precondition was 1963 (first and third quartiles: 1954 and 1969, *N =* 232) for men and 1971 (quartiles: 1965 and 1976, *N =* 1340) for women. Among them 100 and 465 were up to 10 years younger than 55/45 years (men and women, respectively). The mean number of children the respondents had was 2.37 ± 1.18 (mean ± SD, *N =* 1572) in data for the precondition.

**Table 1.** Distribution of the data used in the analysis of the precondition

Variable	Budapest	Whole country
Childhood SES		
Had a very hard time	21	66
Lived worse than average	81	250
Lived at an average standard	423	1061
Lived better than average	82	188
Lived very well	3	7
Sex		
Men	97	232
Women	513	1340
Type of settlement (childhood)		
Village	0	233
Small town	0	214
Medium-sized town	0	185
Large town	0	157
Budapest	610	783

**Table 2.** Sample sizes and sex ratios (number of males/total number of siblings) in the data subsets used for the analysis of the prediction. Mean family (F) and family subunit (S) sex ratios (SRs) and their SDs were calculated for each of the 100 data subsets and also split by childhood socioeconomic status (SES), settlement type during childhood and sex of the respondent. The average values of the mean sex ratios of the 100 data subsets are presented in the table. Median samples sizes (i.e. number of respondents) with their quartiles are also presented for each level of the categorical predictors

		Budapest dataset		Whole country dataset	
Variable	Unit	Median sample size + quartiles, if applicable	Average of mean SRs ± average SDs	Median sample size + quartiles, if applicable	Average of mean SRs ± average SDs
(F) Family SR	F	554	0.49 ± 0.36	1060	0.50 ± 0.37
(S) Family subunit SR	S	554	0.48 ± 0.41	1060	0.49 ± 0.41
By childhood SES					
Worst	F	19 (17, 21)	0.49 ± 0.39	39 (37, 42)	0.50 ± 0.37
	S	19 (18, 20)	0.42 ± 0.41	39 (37, 42)	0.45 ± 0.41
Worse	F	72 (68, 75)	0.44 ± 0.37	169 (165, 176)	0.51 ± 0.37
	S	71 (67, 74)	0.42 ± 0.40	170 (165, 175)	0.49 ± 0.42
Average	F	359 (353, 363)	0.48 ± 0.36	688.5 (682, 692)	0.48 ± 0.37
	S	360.5 (355, 365)	0.48 ± 0.41	687 (679, 692)	0.49 ± 0.41
Better	F	105 (102, 108)	0.54 ± 0.36	163 (159, 168)	0.54 ± 0.37
	S	103 (101, 106)	0.53 ± 0.40	163.5 (160, 169)	0.52 ± 0.41
By type of settlement (childhood)					
Village	F	0		149 (146, 155)	0.52 ± 0.37
	S	0		152 (147, 156)	0.53 ± 0.42
Small town	F	0		139 (134, 144)	0.55 ± 0.37
	S	0		139 (136, 144)	0.54 ± 0.42
Medium-sized town	F	0		125 (121, 128)	0.49 ± 0.37
	S	0		124 (120, 127)	0.49 ± 0.41
Large town	F	0		90 (86, 95)	0.45 ± 0.39
	S	0		90 (86, 94)	0.45 ± 0.42
Budapest	F	554	0.49 ± 0.36	556 (550, 563)	0.49 ± 0.36
	S	554	0.48 ± 0.41	554.5 (549, 560)	0.48 ± 0.41
By sex of respondent					
Men	F	277	0.75 ± 0.25	530	0.77 ± 0.25
	S	277	0.80 ± 0.26	530	0.83 ± 0.25
Women	F	277	0.22 ± 0.25	530	0.23 ± 0.25
	S	277	0.16 ± 0.24	530	0.16 ± 0.23

In the families suitable for the analysis of the prediction of the TWH (2935 and 1545 families in the ‘whole country’ and ‘Budapest’ datasets, respectively, see Figure 1 and Supplementary Table 3) the number of male respondents was low and consequently the mean sex ratios were female biased (proportions of males among the siblings were 0.32 and 0.28 for families and family subunits in the ‘whole country dataset’ and 0.32 and 0.27 for families and family subunits in the ‘Budapest dataset’; see Supplementary Table 3 for mean sex ratios by childhood SES, settlement type and sex of the respondent, calculated from the total dataset). The averages of mean sex ratios of the 100 data subsets that were analysed (see Methods for details) were much more balanced (0.50 and 0.49 for families and family subunits in the ‘whole country dataset’; 0.49 and 0.48 for families and family subunits in the ‘Budapest dataset’; see Table 2 for details).

### TWH precondition

**Figure 2.**
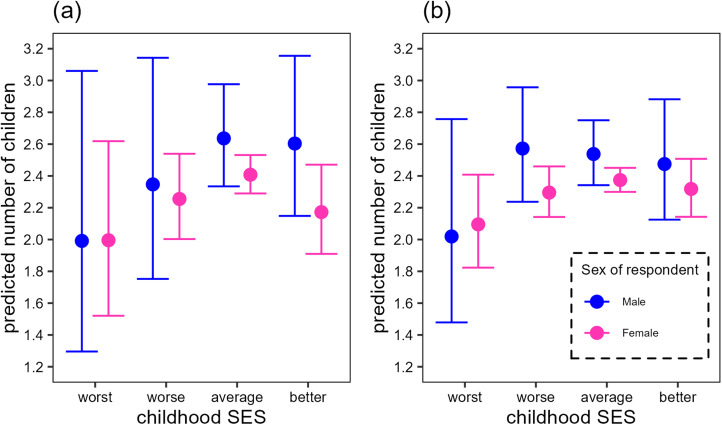
Predicted means (and their 95% confidence intervals) of the number of biological children of Hungarian survey respondents (a) born and living in Budapest (*N =* 610) or (b) from the whole country (*N =* 1572).

**Figure 3.**
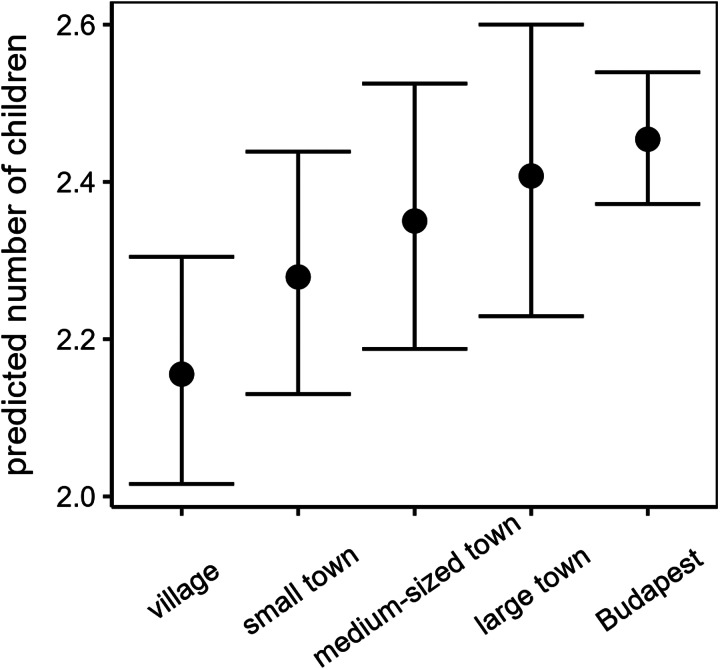
Predicted means (and their 95% confidence intervals) of the number of biological children among Hungarian survey respondents (*N =* 1414) in relation to settlement type during childhood.

We found no clear support for the precondition, because the interaction of sex and childhood SES was not significant irrespective of the dataset used (Table 3 and Figure 2). From the background variables, the number of siblings was a significant predictor only in the ‘whole country dataset’, where the number of children positively correlated with the number of siblings, but not in the ‘Budapest dataset’. When we analysed the ‘whole country dataset’, the type of settlement during childhood was also significant (Table 3, Figure 3). Pairwise comparisons between the category levels of settlement type show that the number of biological children tended to be higher for respondents born in Budapest than villages (villages vs. Budapest: estimate = −0.130, SE = 0.04, *p* = 0.007; Figure 3) when controlling for the other predictors. The other comparisons were not significant.

**Table 3.** The relationship between childhood socioeconomic status and reproductive success among Hungarian survey respondents (a) born and living in Budapest (*N* = 610) and (b) from the whole country (*N* = 1572). Bold indicates type 3 ANOVA results. Italics indicate estimates and standard errors for the intercept, continuous variables and contrast levels with *p*-values from *t*-tests

Response variable: number of children	(a) Budapest dataset					(b) whole country dataset				
Explanatory variables/*contrasts*	*χ^2^*	d.f.	*p*	Estimate	SE	*χ^2^*	d.f.	*p*	Estimate	SE
**Intercept**	**143.29**	**1**	**<0**.**001**	*0*.*767*	*0*.*06*	**315**.**69**	**1**	**<0**.**001**	*0*.*754*	*0*.*04*
**Childhood SES**	**4.09**	**3**	**0**.**254**			**3**.**96**	**3**	**0**.**266**		
*C1: worst – mean (worse, average, better)*			*0*.*169*	*−0*.*185*	*0*.*13*			*0*.*065*	*−0*.*165*	*0*.*09*
*C2: worse – mean (average, better)*			*0*.*478*	*−0*.*062*	*0*.*09*			*0*.*963*	*0*.*002*	*0*.*05*
*C3: average – better*			*0*.*395*	*0*.*058*	*0*.*07*			*0*.*616*	*0*.*025*	*0*.*05*
**Sex**	**0.86**	**1**	**0**.**353**			**0**.**95**	**1**	**0**.**329**		
*Male – mean (female, male)*			*0*.*353*	*0*.*039*	*0*.*04*			*0*.*330*	*0*.*026*	*0*.*03*
**Childhood SES × sex**	**0.79**	**3**	**0**.**852**			**0**.**69**	**3**	**0**.**876**		
*C1, male – C1, mean*			*0*.*693*	*−0*.*053*	*0*.*13*			*0*.*506*	*−0*.*060*	*0*.*09*
*C2, male – C2, mean*			*0*.*581*	*−0*.*048*	*0*.*09*			*0*.*608*	*0*.*024*	*0*.*05*
*C3, male – C3, mean*			*0*.*505*	*−0*.*045*	*0*.*07*			*0*.*990*	*0*.*001*	*0*.*05*
**Number of siblings**	**1.73**	**1**	**0**.**189**	*0*.*028*	*0*.*02*	**6**.**28**	**1**	**0**.**012**	*0*.*032*	*0*.*01*
**Type of settlement**	—	—	—	—	—	**13**.**16**	**4**	**0**.**011**		
*Village – mean*	—	—	—	—	—			*0*.*012*	*−0*.*077*	*0*.*03*
*Small town – mean*	—	—	—	—	—			*0*.*497*	*−0*.*021*	*0*.*03*
*Medium-sized town – mean*	—	—	—	—	—			*0*.*755*	*0*.*010*	*0*.*03*
*Large town – mean*	—	—	—	—	—			*0*.*314*	*0*.*034*	*0*.*03*
	Residual degrees of freedom: 601					Residual degrees of freedom: 1559

### TWH prediction

**Figure 4.**
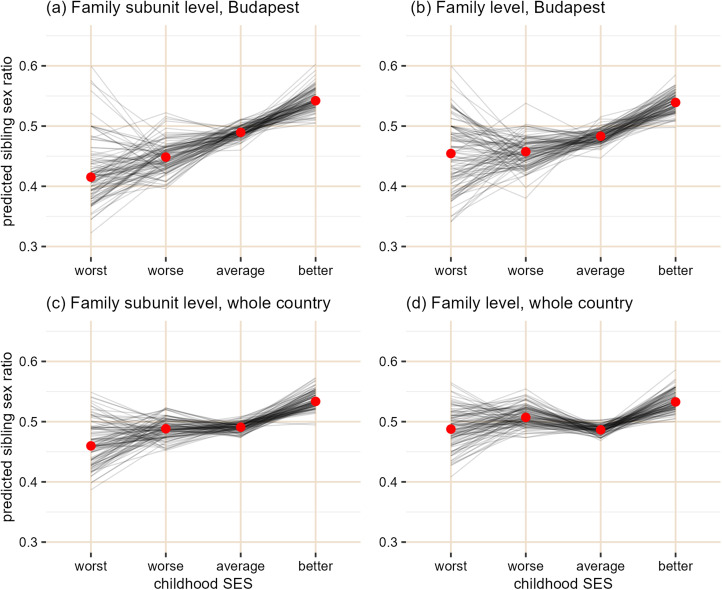
Predicted sibling sex ratios (number of males/total number of siblings) from the 100 generalised linear models on the relationship between childhood SES and sex ratio of siblings in the ‘Budapest’ and ‘whole country’ datasets. One line corresponds to one model's predictions. Red dots indicate the median predicted sex ratio for each level.

Our results varied considerably depending on the level of sex ratio and the dataset used, with a trend to the TWH's predicted direction when the relative living standard estimate was more precise owing to the reduction of spatial (whole country/Budapest) and temporal (family/family subunit) noise in the data (Figure 4). In the 100–100 subsets of the ‘whole country dataset’, there was no clear trend for the relationship between childhood SES and family or family subunit sex ratio (Table 4b and Figure 4c and d). In the ‘Budapest dataset’ the models predicted a slight positive trend, especially in case of the family subunit level analysis that contained members of the family probably born under similar conditions (Table 4a and Figure 4a and b). In the latter case, 33% of the contrasts comparing the ‘worse’ and ‘better’ than average SES groups were significant. This might suggest that family subunits born under better socioeconomic conditions tended to be more male-biased; however, the majority of the omnibus Wald chi-square tests in the ANOVAs were not significant (Table 4).

**Table 4.** *p-*values of the type 3 Wald chi-square tests for SES (in bold) and *p*-values of the contrast ‘worse than average’ – ‘better’ (in italics) from 100 GLMs on the sex ratio of siblings of Hungarian survey respondents (a) born in Budapest and (b) from the whole country while considering settlement type in childhood

Response variable	Family subunit sex ratio	Family sex ratio
Explanatory variables	Median *p* (quartiles)	Median *p* (quartiles)
(a) **Childhood SES**	**0.212** (**0.128, 0.397)**	**0.229** (**0.125, 0.410)**
*‘Worse than average’ – ‘better’*	*0.094* (*0.039, 0.173)*	*0.103* (*0.040, 0.226)*
(b) **Childhood SES**	**0.411** (**0.246, 0.573)**	**0.355** (**0.160, 0.588)**
*‘Worse than average’ – ‘better’*	*0.287* (*0.149, 0.435)*	*0.534* (*0.280, 0.746)*
**Type of settlement**	**0.237** (**0.126, 0.411)**	**0.143** (**0.060, 0.299)**

## Discussion

We examined the TWH in the contemporary Hungarian population. As the assumptions of the TWH are still rarely tested (Douhard, 2017), we aimed to test these before analysing the prediction. The TWH assumes a positive relationship between the mother's and her offspring's condition, and a sex difference in the association of reproductive success and condition. We did not ask directly about the relative living standard of the respondent in adulthood, because it is challenging to construct relative measures of SES that allow reliable comparison of childhood and adulthood conditions. Therefore, we focused on the relationship between childhood SES and sex differences in reproductive success, because, if the assumptions of correlated maternal and offspring's adult condition, and adult condition and reproductive success, are met, this relationship should be observable. Although the difference between the predicted mean reproductive success of men and women tended to increase slightly with SES in the ‘Budapest dataset’ (Figure 2a), and this would be in line with the precondition of the TWH, the confidence intervals were very wide, and the results were very far from being statistically significant. In addition, in the ‘whole country dataset’, we did not find sex difference in the relationship between SES and reproductive success.

In general, in modern contemporary societies, fertility is lower than in natural/historical populations as a result of demographic transitions (Borgerhoff Mulder, 1998), and consequently, the variance of reproductive success is also expected to be smaller within the two sexes (Hruschka & Burger, 2016). Lower variance of reproductive success in both sexes could make it harder to find evidence for the precondition of the TWH. Additionally, sex-selective abortion or especially a bias in the use of induced abortion depending on SES could distort the sex-dependent relationship between reproductive success and SES, and hence mask the effects of the second precondition. Sex-selective abortion has not been reported from Hungary, but the results of Bereczkei and Dunbar (1997) suggest that SES and also sex of the previous offspring might influence the use of induced abortion in Hungary, as the rate of aborted pregnancies differed depending on the sexes of the previous offspring and between the rural and urban populations (note that for the urban population the unemployment rate was lower, and the mean monthly income was higher than for the rural population, suggesting better living standards for the urban population). Also, a negative relationship has been observed between incidence of induced abortion and educational level of women in Hungary (Hungarian Central Statistical Office, 2007, 2018). That is why we decided to analyse the precondition only on data of respondents who were certainly not affected by induced abortion, but still we did not find sex difference in the relationship between SES and reproductive success. We also note that it may have been challenging to detect the interaction because the number of male respondents was rather small.

Here we tested only one of the potential status measures, namely relative living standards, because relative status measures might better predict offspring sex ratio than absolute measures (Douhard, 2017; Lazarus, 2002; Trivers & Willard, 1973). To check whether our estimate of relative SES corresponds to an objective SES proxy and hence our results are comparable with those of other studies using absolute SES measures, we also analysed the relationship between relative living standards of the child at childhood and educational attainment (see Supplementary Information 1). We decided to use this variable as highest educational attainment has been shown to be correlated with material (Edgerton et al., 2012) and psychological well-being (Ross & van Willigen, 1997). Highest educational attainment of the mother significantly correlated both with our measure of childhood SES and the highest educational attainment of the respondent. These results suggest that objective and subjective status measures are somewhat related, and also support that the heredity of status is valid in our study. Overall, our results are in line with the result of Luo et al. (2017) and with most findings of economical and sociological research on social mobility, as it was often found that social status, characterised for example by income, educational attainment occupation, or social class, is heritable (e.g. Bowles and Gintis 2002; Jonsson et al. 2009; Buis 2013; Erola and Jalovaara 2017).

The TWH predicts a positive association between the mother's condition and the sex ratio of her offspring. When we tested the prediction of TWH using the ‘whole country dataset’ and family sex ratios, we found no support for the hypothesis. Given the lack of support for the precondition, one may find this unsurprising. However, the results were somewhat different when, in line with our original intentions, we used the data of families from Budapest. The predicted probabilities of the models on 100 subsets of the data showed a slight trend to the expected direction, but in the majority of the subsets, the omnibus test on the SES categorical variable was not significant. We originally aimed to use the spatially more restricted dataset (i.e. data from Budapest) to reduce noise and thereby increase the chance of detecting even a weak effect of SES. However, not only the spatial heterogeneity of the data may make the detection of sex ratio biases difficult. The reproductive lifespan of humans is very long, and during this, a person's situation can change dramatically: they may experience stressful events such as the death of a close relative, the loss of a job, or conversely, a sudden improvement in the social status may also occur. Experiencing such an event between the births of children could alter the prediction one could make for the offspring sex ratio of a mother, and a family level analysis neglecting this fact may find no correlation between maternal condition and offspring sex even if this relationship exists. Thus, to avoid the noise caused by any substantial change in living standard, we also tested the prediction by analysing the sex ratio of those family members that were born under presumably similar socioeconomic conditions (see Methods). When analysing these family subunit sex ratios in the ‘Budapest dataset’, we found a more pronounced positive trend than for family sex ratios. Thus, we can say that with the reduction of spatial and temporal noise in the data (by using the ‘Budapest dataset’ and family subunit sex ratios, respectively) the observed trend in line with the prediction of the TWH got closer to being significant. Although it does not provide clear evidence for the TWH, it suggests that the predicted effect might be present in the population, but, if present, it is so weak that larger sample sizes are required to detect it.

One may raise the question of how the predicted pattern could emerge if the precondition of the TWH is not confirmed. Hopcroft (2005) noted that the prediction of the TWH may be true even in the absence of sex differences in reproductive success, if humans had physiological adaptations to an evolutionary environment where male and female reproductive success differed in accordance with the second precondition. However, if selection is not acting on sex allocation in the current population, the effects might be small in general, and this may be one of the explanations for the mixed results in the literature, where a large number of papers failed to support the TWH (e.g. Houdek et al., 2020; Kolk & Schnettler, 2016; Morita et al., 2017; Schacht et al., 2019; Schnettler, 2013; Sorokowski et al., 2019; Wu, 2021), while numerous other papers supported it both in contemporary (Bereczkei & Dunbar, 1997; Cameron & Dalerum, 2009; Luo et al., 2017; Pollet et al., 2009; Wallner et al., 2012) and in historical (Li et al., 2022; Mealey & Mackey, 1990) populations. Another explanation for the discrepancy among populations may be that the studied populations differ in several aspects relevant to the TWH. For example, the sex difference in the relationship between reproductive success and SES may depend on the level of polygyny in the study population. In a polygynous population, the sex difference is expected to be large. Accordingly, the hypothesis has been substantially supported in populations in which polygynous marriages occurred (Li et al., 2022; Mackey, 1993; Mealey & Mackey, 1990; Pollet et al., 2009). On the contrary, in socially monogamous societies like Hungary, the variance of the reproductive success is probably less sex-dependent than in polygynous societies, because men's multipartner fertility is limited to sequential polygamy (Forsberg & Tullberg, 1995) or extra-pair relationships. However, the direction of the relationship between multipartner fertility and socioeconomic status may not be unambiguous, for example it was found in a sample of Norwegian men that multipartner fertility was positively associated with both low and high SES (Lappegård & Rønsen, 2013).

In spite of these arguments, there has been evidence in favour of the TWH in monogamous populations as well (see the references above), even from Hungary. Bereczkei and Dunbar (1997), who studied relatively poor sympatric Hungarian and Romani populations, showed that Romanis had more female-biased offspring sex ratios compared with the relatively wealthier Hungarians. Their results are in line with the TWH, although the assumptions of the TWH were not tested directly in their paper. However, it is hard to compare our results and the results of Bereczkei and Dunbar, because the subpopulations studied by them were at the lower end of the socioeconomic scale of Hungary, while we aimed to study the general population, with a possibly wider range of the socioeconomic scale.

There are a few potential limitations of our study which should be mentioned. First, our measure of SES is based on subjective, self-reported information, which, besides its clear advantages (see above), is inherently prone to recall bias by its retrospective nature (Bell & Bell, 2018), and the direction of such a bias is not obvious. Second, as Koziel and Ulijaszek (2001) suggested, TWH probably holds only in those populations where social stratification is sufficiently pronounced. Since we inquired about the self-perceived living standards relative to others, we cannot quantify the actual range of the socioeconomic scale in terms of income or absolute wealth covered by our dataset. It is possible, that despite our intention to target people over a wide range of socioeconomic scale, we could not sample the Hungarian population representatively. For example, those whose living conditions are the worst, are possibly less likely to use the internet (Hungarian Central Statistical Office, 2021; Vallušová et al., 2022), hence they were less likely to participate in our study. Finally, answers to sensitive questions (such as the use of induced abortion) might be affected by social desirability bias (i.e. the respondent answers in such a way that his/her behaviour is likely to be judged favourably by others; Krumpal, 2013), resulting in underreporting the induced abortion rates. However, the risk of social desirability bias was minimised in our case because we guaranteed anonymity and answering these sensitive questions was set to be facultative (Nederhof, 1985).

To sum up, we did not find support for the precondition of the TWH in the contemporary Hungarian population. This might be a result of inadequate sample size, or this might suggest that the selection on SES related sex allocation is currently low or absent. The results concerning the prediction of the TWH were strongly dependent on the dataset used, so further studies are clearly needed. However, focusing on family subunits (i.e. those members of the family that were born under similar conditions), we found a weak sex ratio trend in the predicted direction in the ‘Budapest dataset’ but not at the country level. We think that future studies should test also the preconditions of the TWH, and preferably focus on family subunit sex ratios (as opposed to family sex ratios), because using a point measure of SES and the sex ratio of offspring produced over a longer period with perhaps changing parental SES is less likely to detect sex ratio adjustment even if it is present in the population.

## Supporting information

Sarkadi et al. supplementary material 1 Sarkadi et al. supplementary material

Sarkadi et al. supplementary material 2 Sarkadi et al. supplementary material

Sarkadi et al. supplementary material 3 Sarkadi et al. supplementary material

## Data Availability

The data that support the findings of this study are available on request from the corresponding author, F.S. The data are not publicly available, as we stated in the privacy statement of the questionnaire that the data gathered by us would be used only for scientific purposes.

## Supplementary material

The supplementary material for this article can be found at https://doi.org/10.1017/ehs.2024.39.

## Research transparency and reproducibility

The data that support the findings of this study are available on request from the corresponding author, F.S. The R code used for the statistical analyses and for the figures is available as a Supplementary material.
