# Supplementary material for: Socioeconomic status and sex ratio in the contemporary Hungarian population
Source: Evol Hum Sci. 2024 Oct 30;6:e38. doi: 10.1017/ehs.2024.39 (PMC11658934; doi:10.1017/ehs.2024.39)
Supplement: Sarkadi et al. supplementary material 2 — Sarkadi et al. supplementary material [file S2513843X24000392sup002.docx]

**Supplementary material**

Socioeconomic status and sex ratio in the contemporary Hungarian population

Authors: Fanni Sarkadi, Eszter Szász, Balázs Rosivall

**Supplementary Table 1**

Our questionnaire consisted of three pages (Q1, 2 and 3), of which the third page contained sensitive questions, hence it was set to be facultative. Below we present the questions that we used for data selection and analysis for this manuscript. We also provide information on the type of answer and answer options (if applicable). Note that some questions were conditional and appeared depending on the answers to previous questions (see the “criteria” column).

| CRITERIA |  | QUESTION | TYPE OF ANSWER | ANSWER OPTIONS |
| --- | --- | --- | --- | --- |
|  | Q1.0 | I have read the privacy notice. I would like to fill in the questionnaire. | checkbox | Yes |
|  | Q1.1a | To your knowledge, have any of your siblings already filled in the questionnaire? | radio button | Yes / No |
| Q1.1a “Yes” | Q1.1b | Please give the unique code of your sibling | text box |  |
|  | Q1.2a | To your knowledge, has your spouse already filled in the questionnaire? | radio button | Yes / No |
| Q1.2a “Yes” | Q1.2b | Please give the unique code of your spouse | text box |  |
|  |  | **Please provide the following information** |  |  |
|  | Q1.3 | Sex | radio button | Male / Female |
|  | Q1.4 | Year of birth | text box |  |
|  | Q1.5a | Do you have any full sibling? | radio button | Yes/No |
| Q1.5a “Yes” | Q1.5b | Please give the number of your full siblings | text box |  |
|  | Q1.6 | Country where you are living | radio button | Hungary/Other |
| Q1.6 “Hungary” | Q1.7a | Current residency | radio button | Budapest / Other |
| Q1.7a “Other” | Q1.7b | Please choose the type of your residency | radio button | village (<5000 residents) / small town (5-20 000) / medium-sized town (20-100 000) / large town (>100 000) |
| Q1.7a “Other” | Q1.7c | Please choose your county of residence | roll down menu |  |
|  | Q1.8a | Residency at the time of your birth (the settlement where your family lived at the time of your birth) | radio button | Budapest / Other Hungarian settlement / Settlement abroad |
| Q1.8a “Other Hungarian settlement” | Q1.8b | Please choose the type of residency | radio button | village (<5000 residents) / small town (5-20 000) / medium-sized town (20-100 000) / large town (>100 000) |
| Q1.8a “Other Hungarian settlement” | Q1.8c | Please choose the county where your family lived | roll down menu |  |
|  | Q1.9 | What was your family's standard of living during your childhood? Try to recollect the period before your age of 10 (If your standard of living changed significantly during this period, please, choose the answer that corresponds to the earliest years). | radio button | We had a very hard time / We lived worse than average / We lived at an average standard / We lived better than average / We lived very well |
| Q1.3 “Male” | Q1.10a | In your opinion, how did you live during your adult life (18-55 years) compared to your childhood living standard (before your age of 10)? (If your standard of living changed significantly during your adult life, please, choose the answer that depict the longest period of time.) | radio button | Substantially worse / Somewhat worse / More or less the same / Somewhat better / Substantially better |
| Q1.3 “Female” | Q1.10b | In your opinion, how did you live during your adult life (18-45 years) compared to your childhood living standard (before your age of 10)? (If your standard of living changed significantly during your adult life, please, choose the answer that depict the longest period of time.) | radio button | Substantially worse / Somewhat worse / More or less the same / Somewhat better / Substantially better |
|  | Q1.11 | Your highest educational attainment | radio button | Did not complete the primary school / primary school / vocational school (without high school diploma) / high school diploma / college or bachelor’s degree / university (master degree) |
|  | Q1.12 | Your father’s highest educational attainment | radio button | Did not complete the primary school / primary school / vocational school (without high school diploma) / high school diploma / college or bachelor’s degree / university (master degree) |
|  | Q1.13 | Your mother’s highest educational attainment | radio button | Did not complete the primary school / primary school / vocational school (without high school diploma) / high school diploma / college or bachelor’s degree / university (master degree) |
|  | Q1.14 | Family status | radio button | Single / In a relationship |
|  | Q1.15a | Do you have any biological children? | radio button | Yes / No |
| Q1.15a “Yes” | Q1.15b | Number of biological children from all your relationships (If you and your partner are currently expecting a child, please include the foetus!) | roll down menu |  |
| Q1.15a “Yes” | Q1.15c | How many of these are sons? | text box |  |
| Q1.15a “Yes” | Q1.15d | How many of these are daughters? | text box |  |
| Q1.15a “Yes” | Q1.15e | How many of these are foetuses of unknown sex? | text box |  |
| Q1.14 “In a relationship” | Q1.16 | Would you and your current partner like to have more children? | radio button | Yes / No / We haven’t thought about it |
| Q1.14 “In a relationship” | Q1.17 | Do you think you and your partner will still be together in 10 years? | radio button | Yes / No / I don’t know |
|  |  | **Please provide the following information about your full siblings**  **/***The following section appeared as many times as the number of siblings of the respondent/* |  |  |
|  | Q2.18 | Sex | radio button | Male / Female |
|  | Q2.19 | Year of birth | text box |  |
|  | Q2.20a | Place of birth (the place where your family lived when your brother or sister was born) | radio button | Budapest / Other Hungarian settlement/Abroad |
| Q2.20a “Other Hungarian settlement” | Q2.20b | Please choose the type of residency | radio button | village (<5000 residents) / small town (5-20 000) / medium-sized town (20-100 000) / large town (>100 000) |
|  | Q2.21a | Does your sibling have any biological children? | radio button | Yes / No |
| Q2.21a “Yes” | Q2.21b | Number of biological children (If your sibling and his/her partner are expecting a child, please, include the foetus too!) | roll down menu |  |
| Q2.21a “Yes” | Q2.21c | How many of these are sons? | text box |  |
| Q2.21a “Yes” | Q2.21d | How many of these are daughters? | text box |  |
| Q2.21a “Yes” | Q2.21e | How many of these are foetuses of unknown sex? | text box |  |
|  |  | **The following questions apply to the children of your biological parents (you and your full siblings). The oldest child (including yourself) is the first.** |  |  |
|  | Q2.22 | Please tick those events that have happened between the birth of your parents’ *X*th and *X*+1th children, and significantly influenced your family’s life.  *The same question was asked for all consecutive (Xth and X+1th) children* | checkbox | Death of a close relative (grandparent, parents’ siblings, your siblings) / Serious accident of a close relative / Serious but transient or chronic illness of a close relative / Mother’s failed pregnancy (miscarriage) / Significant change in the family’s financial situation (either positive or negative) / Divorce of grandparents / I’m not aware of any of these events |
|  | Q2.23a | Are there twins among your siblings? | radio button | No / Yes, identical twins / Yes, fraternal twins / Yes, identical and fraternal twins as well |
| Q2.24a “Yes, (…)” | Q2.23b | Type the birth year(s) of the twins (if there are multiple twins in the family, use comma between the years) | text box |  |
|  | Third page | **Please answer the questions below (optional)** |  |  |
| Q1.3 “Female” | Q3.24 | Have you had an unsuccessful pregnancy (miscarriage)? | radio button | Yes / No |
| Q1.3 “Female” | Q3.25 | Have you had an induced abortion during any of your pregnancies? | radio button | Yes / No |
| Q1.3 “Male” | Q3.26 | To your knowledge, have any of your sexual partners (including long-term partners) had an induced abortion during a pregnancy where you could be the father? | radio button | Yes / No |

**Supplementary Information 1**

**Correlation between highest educational attainment and relative SES**

It has been shown that education level fundamentally influences material well-being (reviewed by Edgerton et al., 2012). In addition, educational attainment is associated with subjective well-being as well, as data of the representative Household Budget and Living Condition Survey conducted by the Hungarian Central Statistical Office showed a positive trend between educational attainment and two aspects of subjective well-being, namely, overall satisfaction with life and satisfaction with the household’s financial situation (see the link: statinfo.ksh.hu/Statinfo/QueryServlet?openLink=e2c71d3f38f35225bdfbf1665f534bae02a0ff927f4c170d14aa4a607df0ab0e). Therefore, we validated the use of our estimate of childhood SES by checking whether relative living standard during childhood was correlated with educational attainment of the mother. We also tested the association of the respondent’s and his/her mother’s education level. As childhood SES and educational attainments of the mother and the respondent are ordinal variables, we used Kendall’s Tau correlation with function “cor.test” from package “stats” (R Core Team, 2023).

The database for these tests were somewhat larger than the dataset for the precondition: since the use of induced abortion was not an issue here, the sample size was 2307 (see Figure 1 in the main text for exclusion criteria and Supplementary Table 2 for detailed sample sizes).

Childhood SES and the mother’s educational level positively correlated (Z = 9.71, tau = 0.17, p<0.001, N = 2307; Supplementary Figure 1b). We found support for the heredity of status in the Hungarian population, because the respondent’s own and the mother’s educational attainment positively correlated (Z = 17.93, tau = 0.31, p<0.001, N = 2307; Supplementary Figure 1a).

**Supplementary Table 2**

***Sample sizes for the rang correlation test on educational attainment and childhood SES***

| **variable** | | **Whole country** |
| --- | --- | --- |
| childhood SES | |  |
|  | very hard time | 105 |
|  | worse than average | 373 |
|  | average | 1524 |
|  | better than average | 290 |
|  | very well | 15 |
| educational attainment of the respondent | |  |
|  | did not complete primary school | 0 |
|  | primary school | 7 |
|  | vocational school without high school diploma | 70 |
|  | high school diploma | 479 |
|  | college or bachelor’s degree | 808 |
|  | university (master degree) | 943 |
| educational attainment of the respondent’s mother | | |
|  | did not complete primary school | 42 |
|  | primary school | 330 |
|  | vocational school without high school diploma | 374 |
|  | high school diploma | 789 |
|  | college or bachelor’s degree | 431 |
|  | university (master degree) | 341 |

**Supplementary Figure 1**

***The relationship between (a) childhood SES and mother’s educational attainment, and (b) respondent’s and mother’s educational attainment among Hungarian survey respondents (N = 2026).*** ***Pink numbers indicate the sample size of each education level category.***


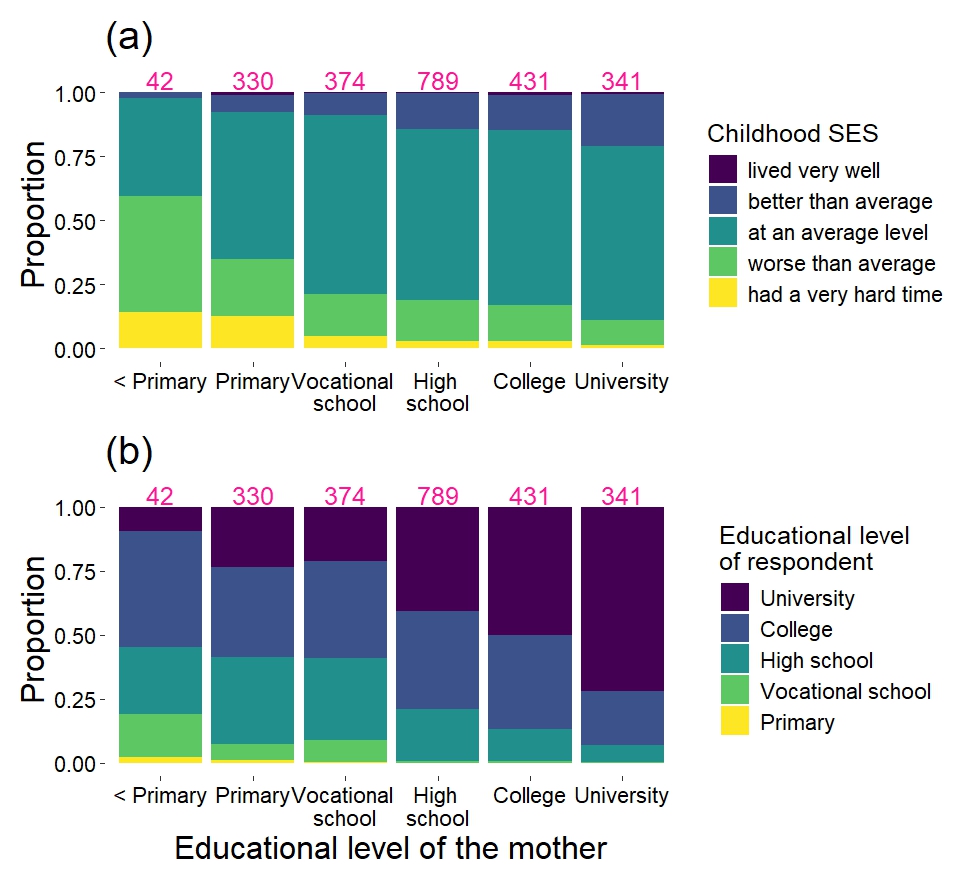


**Supplementary Information 2**

**Age distribution of respondents and reproductive timing in Hungary**

Among the respondents involved in the analysis of the precondition (N = 1414), 202 were men (89 was above age 45, but not older than 55; 113 was above age 55) and 1212 were women (75 was above age 35, but not older than 40; 351 was above 40, but not older than 45; 786 was above age 45). These respondents are unlikely to have more children later in their life than reported. First, based on data of the Hungarian Central Statistical Office, the number of live births per 1,000 women decreases from the age of 30 onwards, and the cumulative number of live births per 1,000 women aged above 40 was 35.2, while this cumulative number for women aged above 45 was only 1.4 in 2018 (see the link (in Hungarian): <https://www.ksh.hu/docs/hun/xftp/idoszaki/szuletesek_termekenyseg/index.html#aszletseksatermkenysgalakulsahosszabbtvonmeghatrozzaanpessgszmvltozst>). The number of live births per 1,000 men is decreasing from approximately 34 years, and only 5% of live births in 2016 had a father aged 45 or older (Makay & Spéder 2018, in Demographic Portrait 2018, HCSO Hungarian Demographic Research Institute, link (in Hungarian): <https://demografia.hu/kiadvanyokonline/index.php/demografiaiportre/article/view/2741/2638>). Therefore, above the age of 45/55 (women/men), it is highly unlikely that a respondent will have a child. Nevertheless, we have excluded those who responded that they were still planning to have more children. As for respondents up to 10 years younger than the criterion, we only included those who stated to be in a stable relationship and not planning to have children.

**Supplementary Table 3**

***Family (F) and family subunit (S) sex ratios (mean plus standard deviation) by childhood SES, settlement type during childhood and sex of the respondent for the whole dataset. Note that the GLMs for the prediction were run on subsets of the data, because of the substantial difference in the number of male and female respondents (see Methods).***

|  |  | Budapest dataset | | Whole country dataset | |
| --- | --- | --- | --- | --- | --- |
| **variable** | | **sample size** | **mean ± SD** | **sample size** | **mean ± SD** |
| (F) family and (S) family subunit SR | | 1545 | F 0.32 ± 0.32  S 0.27 ± 0.34 | 2935 | F 0.32 ± 0.33  S 0.28 ± 0.35 |
| by childhood SES | |  |  |  |  |
|  | had a very hard time | 54 | F 0.31 ± 0.33  S 0.22 ± 0.34 | 111 | F 0.32 ± 0.32  S 0.25 ± 0.34 |
|  | lived worse than average | 225 | F 0.30 ± 0.31  S 0.34 ± 0.32 | 469 | F 0.33 ± 0.33  S 0.28 ± 0.35 |
|  | lived at an average level | 1012 | F 0.32 ± 0.32  S 0.28 ± 0.34 | 1943 | F 0.32 ± 0.32  S 0.28 ± 0.35 |
|  | lived better than average | 244 | F 0.35 ± 0.34  S 0.30 ± 0.38 | 390 | F 0.35 ± 0.34  S 0.29 ± 0.36 |
|  | lived very well | 10 | F 0.38 ± 0.31  S 0.33 ± 0.33 | 22 | F 0.40 ± 0.33  S 0.38 ± 0.38 |
| by type of settlement (childhood) | |  |  |  |  |
|  | village | 0 |  | 401 | F 0.34 ± 0.33  S 0.30 ± 0.37 |
|  | small town | 0 |  | 375 | F 0.36 ± 0.34  S 0.30 ± 0.37 |
|  | medium-sized town | 0 |  | 347 | F 0.32 ± 0.33  S 0.27 ± 0.35 |
|  | large town | 0 |  | 267 | F 0.28 ± 0.33  S 0.24 ± 0.34 |
|  | Budapest | 1545 | F 0.32 ± 0.32  S 0.27 ± 0.34 | 1545 | F 0.32 ± 0.32  S 0.27 ± 0.34 |
| by sex of respondent | |  |  |  |  |
|  | men | 277 | F 0.75 ± 0.25  S 0.80 ± 0.26 | 530 | F 0.77 ± 0.25  S 0.83 ± 0.25 |
|  | women | 1268 | F 0.22 ± 0.25  S 0.16 ± 0.24 | 2405 | F 0.23 ± 0.25  S 0.16 ± 0.23 |
